# The association between ethnicity, socioeconomic position and outcomes following initiation of TNF inhibitors in juvenile idiopathic arthritis: results from the UK JIA Biologics Register

**DOI:** 10.1093/rheumatology/keag318

**Published:** 2026-06-19

**Authors:** Richard P Beesley, Eileen Baildam, Michael W Beresford, Sharon Douglas, Taunton R Southwood, the UK JIA Biologics Register, Devesh Mewar, Helen Marzo-Ortego, Flora McErlane, Ben Parker, Elizabeth MacPhie, Srinivasan Venkatachalam, Sharmin Nizam, Ian Gaywood, Rachel Tattersall, Niki Erb, Abbas Ismail, Coziana Ciurtin, Debajit Sen, Professor Robert Moots, Elizabeth Murphy, Easwaradhas Gladston-Chelliah, Mark Quinn, Kate Armon, Michael Beresford, Liza McCann, Gillan Bask, Lorna Walding, Susan Marchant, Nagui Gendi, Eslam Al-Abadi, Charlene Bass-Woodcock, Athimalaipet Ramanan, Louise Walker, John Bourne, Bridget Oates, Vicky Ohlsson, David James, Nick Wilkinson, Kathy Bailey, Flora McErlane, Ovgu KulCinar, Gill Pountain, Mitra, Mark Wood, Arani Sridhar, Karen Davies, Kate Armon, Rachel Jeffery, Imogen Norton, Kathy Bailey, Afraa Al-Sabbagh, Fouz Rahmeh, Joanne Borbone, Alison Leak, Rangaraj Satyapal, Kishore Warrier, Gulshan Malik, Richard Bowker, Nigel Osborne, Catriona Anderson, Jo Walsh, Alice Chieng, Richard Brough, Jonathon Packham, Alex Tabor, Daniel Hawley, Daniel Hawley, Alice Leahy, Rajesh Rawlani, Jon Packham, Jo Walsh, Rosemary Waller, Karen Davies, Willam Coles, Jeremy Camilleri, Lisa Bray, Kimme L. Hyrich, Jenny H. Humphreys, Lianne Kearsley-Fleet

**Affiliations:** Centre for Epidemiology, Centre for Musculoskeletal Research, The University of Manchester, Manchester Academic Health Science Centre, Manchester, United Kingdom; The Alexandra Hospital, Cheadle, Manchester, United Kingdom; Department of Paediatric Rheumatology, Alder Hey Children’s NHS Foundation Trust, Liverpool, United Kingdom; Institute of Life Course and Medical Sciences, University of Liverpool, Liverpool, United Kingdom; Scottish Network for Arthritis in Children (SNAC), Isle of Arran, United Kingdom; Institute of Child Health, University of Birmingham, Birmingham, United Kingdom; Centre for Epidemiology, Centre for Musculoskeletal Research, The University of Manchester, Manchester Academic Health Science Centre, Manchester, United Kingdom; National Institute of Health Research Manchester Biomedical Research Centre, Manchester University NHS Foundation Trust, Manchester, United Kingdom; Centre for Epidemiology, Centre for Musculoskeletal Research, The University of Manchester, Manchester Academic Health Science Centre, Manchester, United Kingdom; National Institute of Health Research Manchester Biomedical Research Centre, Manchester University NHS Foundation Trust, Manchester, United Kingdom; Centre for Epidemiology, Centre for Musculoskeletal Research, The University of Manchester, Manchester Academic Health Science Centre, Manchester, United Kingdom

**Keywords:** ethnicity, socioeconomic position, juvenile idiopathic arthritis, disease activity, treatment persistence, biologic DMARD

## Abstract

**Objectives:**

Health outcomes in children and young people are known to vary by ethnicity and socioeconomic position. In juvenile idiopathic arthritis (JIA), it is unclear whether this relates to differential changes following one of the most common treatments, TNF-inhibitors (TNFi). This study investigated these factors, disease activity and treatment persistence following initial TNFi therapy in patients with JIA in the UK.

**Methods:**

Patients with non-systemic JIA in the UK JIA Biologic Register starting their first TNFi biologic were included. Outcomes included change in disease activity between start of TNFi and 6 months, measured by JADAS-71. Multivariable linear regression was used to assess the association between ethnicity or socioeconomic position and change in JADAS. Treatment persistence was analysed using Kaplan–Meier estimates. Cox proportional hazards models compared TNFi drug persistence by ethnic group and by socioeconomic position.

**Results:**

A total of 1641 patients were included: 67% female, 90% White ethnic group (6% Asian, 2% Black, 2% Mixed), 25% in the most deprived socioeconomic group. JADAS-71 improved for all ethnic and socioeconomic groups by 6 months, with no difference in improvement by group. The proportion of patients remaining on TNFi at 12 months (67%) and the likelihood of stopping was similar between all ethnic and socioeconomic groups.

**Conclusion:**

Outcomes following TNFi initiation are similar between ethnic and socioeconomic groups. Based on the results of this study, ethnicity and socioeconomic position do not appear to be associated with differential change in disease activity, and there is no evidence that the effects of socioeconomic position are moderated by ethnicity or vice versa.

## Graphical abstract

**Graphical Abstract:**
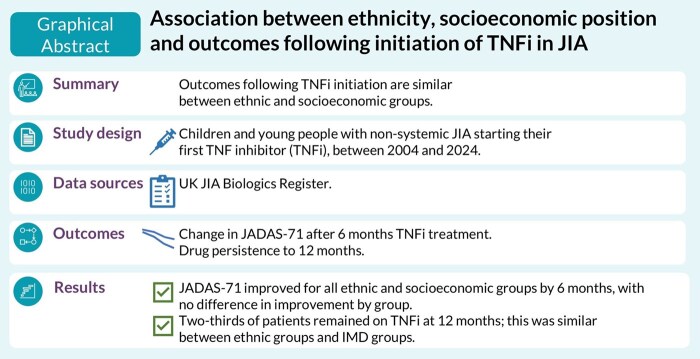

Rheumatology key messages

- JIA disease activity improved over 6 months following TNFi initiation for all ethnic groups.
- The proportion of JIA patients remaining on TNFi at 12 months was similar between ethnic groups.
- Ethnicity does not appear to be associated with differential change in JIA disease activity.

## Introduction

Juvenile idiopathic arthritis (JIA) is a group of related chronic autoimmune conditions characterized by persistent joint inflammation of unknown cause with onset before the age of 16 years [1]. JIA is diagnosed in around one in 1600 children and young people, with potential variances in incidence and prevalence between different ethnic groups [2].

JIA can lead to long-term consequences including joint damage, chronic pain and lifelong disability. These effects may significantly impact both physical and mental well-being, as well as social and economic prospects [3, 4]. However, contemporary treatment approaches have generally improved patient outcomes.

Treatment of JIA in the United Kingdom (UK) usually follows National Health Service (NHS) guidelines [5]. For children and young people with JIA for whom conventional synthetic disease-modifying antirheumatic drugs (DMARDs) do not adequately control disease, treatment with biological therapies may commence. Following the treatment pathway, for most patients, the first class of biologic used is a TNF inhibitor (TNFi). Treatment persistence is a composite real-world indicator for long-term therapeutic success, reflecting both the efficacy and tolerability of a medication.

Ethnicity and socioeconomic position have been associated with differences in access to healthcare, disease activity and disease outcomes in JIA and other conditions [6–11]. However, none have looked at the association of ethnicity and/or socioeconomic position with changes in disease activity in individuals with JIA after starting biologic therapies. Given these factors may affect outcomes generally, understanding whether they are directly associated with outcomes following initiation of biologic therapies is important in shaping appropriate care for patients.

This analysis aimed to investigate changes in disease activity and treatment persistence following initial treatment with TNFi in children and young people with JIA in the UK, by ethnic group and socioeconomic position.

## Methods

### Study design

This analysis used data collected through the UK JIA Biologics Register, which consists of the British Society for Paediatric and Adolescent Rheumatology Etanercept Cohort (BSPAR-ETN) and the Biologics for Children with Rheumatic Diseases (BCRD) studies. These studies share identical methodologies and data collection processes, as previously presented [12]. To summarize, since 2004 for etanercept and since 2010 for all other advanced therapies, children with JIA have been recruited at the point of starting a biologic or JAK inhibitor therapy for their JIA, in order to investigate the safety and effectiveness of these therapies.

### Data collection

Baseline data, including patient demographics, JIA ILAR category [1], time since diagnosis, current disease activity measured using the JIA core outcome variables [13] and a pain visual analogue scale (VAS), and details of previous and current antirheumatic therapies were collected at the start of TNFi treatment.

Follow-up data were collected after 6 months, and 12 months, and then annually. This included changes to antirheumatic drug therapy including start/stop dates and reasons, the most recent recorded JIA core outcome variables, and adverse events. All data were captured from patient medical records at each time point as part of routine care, with no additional study visits for the purposes of data collection.

### Ethnicity

Self-declared ethnicity was collected at recruitment, using the ethnicity category declared by the patient/guardian to the hospital and recorded in their medical record. For all analyses, ethnicity was grouped according to the five first-tier ethnic group categories—White, Asian, Black, Mixed and Other—according to the Office for National Statistics (ONS) England ethnic group classification [14].

### Indices of deprivation

Patients were assigned to a nationwide deprivation rank using nationally published Indices of Multiple Deprivation (IMD) [15], which is a composite indicator of relative deprivation across multiple domains, based on home address postcode. IMD ranks are defined and allocated by the national statistics agency of each nation of the UK. The calculation of IMD scores differs between nations in the UK, therefore quintiles were determined separately for England (based on 2019 scores), Scotland (based on 2012 scores) and Wales (based on 2014 scores), then combined into an overall IMD quintile score; no patients were from Northern Ireland. IMD was grouped into a dichotomous variable; patients living in the most deprived quintile of addresses compared with all other patients.

### Patient selection

This analysis included JIA patients who were starting their first biologic, which was a TNFi, between 1 January 2004 and 5 April 2024, registered within 6 months of starting their TNFi for whom ethnicity was recorded and at least one follow-up form had been completed. Data were extracted on 20 September 2024, and all data returned before this date was included. Patients with systemic JIA were excluded as TNFi are no longer considered the first line of biologic treatment for this subtype.

### Analysis

Demographics and disease characteristics are presented at start of TNFi therapy for the total study population, and by ethnic group and IMD group. Counts of fewer than five patients are suppressed due to requirements of non-disclosure under GDPR.

#### Outcomes following initial treatment with first TNFi

For analyses of change in disease activity, patients were additionally required to have at least one measure of disease activity recorded no more than 6 months before commencing TNFi treatment as part of their baseline assessment.

The primary outcome was the mean change in Juvenile Arthritis Disease Activity Score [16], based on 71 joint-count (JADAS-71) between TNFi treatment initiation and after 6 months of follow-up; outcomes were presented regardless of whether a patient remained on treatment. The JADAS-71 is a composite score, calculated by summing the Active Joint Count (AJC), Physician’s Global Assessment of overall disease activity (PGA), Patient/Parent Global Evaluation of overall wellbeing (PGE) and normalized erythrocyte sedimentation rate (ESR), giving a final total score between 0 (low disease activity) and 101 (very high disease activity).

Secondary outcomes included disease activity outcomes after 6 months of treatment: (i) the proportion of patients attaining minimal disease activity (MDA, Supplementary Table S1) [17]; (ii) proportion of patients attaining ACR paediatric 30%, 50%, 70% and 90% responses (ACR-Pedi-30/50/70/90), modified to allow for low baseline disease activity scores (Supplementary Table S2); (iii) mean change in clinical JADAS-71 (cJADAS-71) – composite score excluding ESR [18]; (iv) mean change in each of AJC, limited joint count (LJC), PGA, PGE, Childhood Health Assessment Questionnaire of functional ability (CHAQ) [19, 20], ESR and patient/parent reported pain (scale 0–10).

The association between ethnicity, IMD and change in JADAS-71 from start of TNFi therapy to after 6 months of treatment was analysed using multivariable linear regression, adjusted for gender, age, time between diagnosis and commencement of TNFi treatment, year of treatment start, ILAR category, history of uveitis, use of glucocorticoids at start of treatment and baseline JADAS-71; these covariates were chosen *a priori* in advance. An interaction term between ethnic group and IMD was included in the model.

#### Persistence of TNFi treatment

All patients were included in analysis of drug persistence over the first 12 months of treatment with a TNFi.

Kaplan–Meier estimates were used to analyse treatment persistence of a patient’s first TNFi. Patients entered the model at the start of TNFi treatment and were considered to remain on drug until stopping their first TNFi for any reason other than for remission, date of last follow-up, death or 12 months from commencement of TNFi, whichever was earliest. Patients were censored if they stopped TNFi reporting remission as the stop reason. Kaplan–Meier curves of TNFi persistence up to 12 months by ethnic group and by IMD are presented, and the proportion of patients remaining on TNFi at 12 months was estimated. Cox proportional hazards models were used to compare TNFi drug persistence by ethnic group and by IMD, adjusted for gender, age, time between diagnosis and commencement of TNFi treatment, history of uveitis, ILAR category and an interaction term between ethnicity and IMD. A sensitivity analysis of only patients starting initial TNFi from 2010 onwards was completed to account for the limited availability and choice of drug of biologic therapies prior and the impact that may have on treatment persistence.

### Missing data

IMD data were not available for all participants; analyses including IMD as a covariable were therefore restricted to participants with recorded IMD values. Multiple imputation using chained equations (72 datasets) was used to account for missing disease activity and covariate data for patients where IMD was available. Complete variables included in the imputation model included age, gender, ethnic group, IMD, history of uveitis, drug and ILAR category. Imputed disease activity variables included AJC, LJC, PGA, PGE, CHAQ, ESR and pain, at both baseline and at six months. JADAS-71 and cJADAS-71 (at baseline and six months) and change in JADAS-71 and cJADAS-71, MDA and ACR-Pedi-30/50/70/90 were calculated using imputed variables.

Analysis was performed using Stata Version 14.0.

### Patient involvement

Prior to this analysis the authors discussed the proposed project with parents of children and young people with JIA, from a diverse range of ethnic backgrounds. Their role was to help identify priorities for analysis and develop the analysis plan. Parent involvement was conducted through online discussion groups and interviews with the lead author, with priorities for analysis collated into the initial analysis plan which parents then reviewed and revised. This analysis is thus informed by the needs of patients and families, reflecting the identities and interests of a diverse population. Whether disease outcomes in JIA are associated with ethnicity and social determinants of health was reported to be a priority area to investigate by families. Patient involvement supported the development of both this analysis and a previous analysis regarding outcomes following methotrexate [21].

### Ethical approval

Both BCRD and BSPAR-ETN have UK Health Research Authority ethical approval and all parents (and, where appropriate, patients) gave informed written consent in accordance with the Declaration of Helsinki. No further ethics approval was required to undertake the current analysis.

## Results

### Baseline characteristics

A total of 1641 children and young people with JIA starting their first biologic as a TNFi met the inclusion criteria and were included in this analysis (Supplementary Fig. S1). Of these, 67% were female, 90% were in the White ethnic group (6% Asian, 2% Black, and 2% Mixed ethnicity), and 25% were in the most deprived IMD quintile (Table 1). The proportion of patients of White ethnicity was slightly higher than national population estimates by ethnicity for children under the age of 16 [22] (90% in this analysis, compared with 73% of the population and 83% of incident JIA cases; Supplementary Table S3). At the start of TNFi treatment, the median age was 11 years (inter-quartile range, IQR: 7–14 years) and median time between diagnosis and start of TNFi was 2 years (IQR: 1–5). The most frequent ILAR category for all ethnic groups was rheumatoid factor (RF) negative polyarthritis (37% of all patients, range 30–37% by ethnic group). The majority of patients started etanercept (68%) followed by adalimumab (29%) with fewer starting on infliximab (4%); this did not differ by ethnic group.

**Table 1:** Baseline characteristics of 1641 children and young people with JIA commencing first TNFi (included in the drug persistence analysis).

Characteristic		Whole cohort	Ethnic group				IMD group	
			White	Asian	Black	Mixed	Most deprived quintile	All others
N (row %)		1641	1481 (90)	97 (6)	27 (2)	36 (2)	332 (25)	972 (75)
Gender, *n* (%)	Male	538 (33)	483 (33)	29 (30)	12 (44)	14 (39)	106 (32)	314 (32)
	Female	1103 (67)	998 (67)	68 (70)	15 (56)	22 (61)	226 (68)	658 (68)
Age at start of TNFi	Median (IQR)	11 (7, 14)	11 (7, 14)	12 (8, 14)	12 (8, 14)	10 (5, 14)	10 (7, 13)	11 (7, 14)
Disease duration (time between diagnosis and commencement of TNFi), years	Median (IQR)	2 (1, 5)	2 (1, 5)	2 (1, 4)	2 (1, 6)	2 (1, 3)	2 (1, 5)	2 (1, 5)
TNFi, *n* (%)	Etanercept	1114 (68)	1010 (68)	64 (66)	16 (59)	24 (67)	233 (70)	594 (61)
	Adalimumab	468 (29)	423 (29)	26 (27)	11 (41)	8 (22)	84 (25)	340 (35)
	Infliximab	59 (4)	48 (3)	7 (7)	0 (0)	4 (11)	15 (5)	38 (4)
Start year of TNFi, *n* (%)	Pre-2010	500 (30)	468 (32)	23 (24)	4 (15)	5 (14)	81 (24)	199 (20)
	2010–2015	521 (32)	453 (31)	38 (39)	10 (37)	20 (56)	126 (38)	339 (35)
	2016 onwards	620 (38)	560 (38)	36 (37)	13 (48)	11 (31)	125 (38)	434 (45)
Indices of Multiple Deprivation (IMD) quintile, *n* (%)	1—Most deprived	332 (25)	276 (23)	33 (42)	12 (62)	10 (34)	—	—
	2	220 (17)	193 (16)	20 (25)	<5	< 5	—	—
	3	264 (20)	245 (21)	12 (15)	<5	6 (21)	—	—
	4	234 (18)	218 (19)	<5	<5	<5	—	—
	5—Least deprived	254 (19)	243 (21)	<5	<5	8 (28)	—	—
ILAR category, *n* (%)	Persistent oligo	195 (12)	168 (11)	17 (18)	5 (19)	5 (14)	38 (11)	135 (14)
	Oligo extended	336 (20)	319 (22)	6 (6)	5 (19)	6 (17)	80 (24)	192 (20)
	Poly RF-	601 (37)	546 (37)	35 (36)	8 (30)	12 (33)	108 (33)	360 (37)
	Poly RF+	156 (10)	133 (9)	16 (16)	<5	<5	30 (9)	88 (9)
	Psoriatic	108 (7)	100 (7)	6 (6)	<5	<5	31 (9)	55 (6)
	Enthesitis-related	188 (11)	168 (11)	9 (9)	5 (19)	6 (17)	32 (10)	121 (12)
	Undifferentiated	57 (3)	47 (3)	8 (8)	<5	<5	13 (4)	21 (2)
History of chronic anterior uveitis at start of treatment, *n* (%)	Yes	303 (20)	268 (19)	20 (22)	8 (32)	7 (21)	58 (18)	194 (21)
	No	1222 (80)	1109 (81)	69 (78)	17 (68)	27 (79)	256 (82)	726 (79)

IMD: Index of Multiple Deprivation; IQR: interquartile range; time to registration: time (years) between diagnosis of JIA and registration and commencement of treatment.

A total of 1418 patients met the additional inclusion criteria for analyses of disease activity (67% female; 90% White ethnic group, 6% Asian, 2% Black, 3% Mixed ethnicity; 25% in the most deprived IMD quintile; Supplementary Table S5). This cohort appeared broadly similar to the whole group of 1641 patients included in the drug persistence analysis in all demographic features. Missingness is presented in Supplementary Table S6.

### Outcomes following initial treatment with first TNFi

Following treatment with TNFi for 6 months, disease activity had improved for all ethnic groups (Table 2). Mean change in JADAS-71 at six months (95% CI) was broadly similar for all ethnic groups: –8.9 units (–9.5, –8.3) for patients of White ethnicity, –6.8 (–9.0, –4.7) for patients of Asian ethnicity, –12.7 (–19.3, –6.2) for patients of Black ethnicity and –7.9 (–10.7, –5.2) for patients of Mixed ethnicity. The two IMD dichotomized groups (see above) both showed similar improvement in JADAS: most deprived quintile –7.7 (–8.7, –6.5), all others –8.4 (–9.1, –7.7). Both unadjusted and adjusted relative mean change in JADAS-71, relative to White ethnicity, were similar for Asian, Black and Mixed children and young people, and similar for both IMD groups.

**Table 2:** Change in JADAS-71 between baseline and six months after initiation of TNFi treatment amongst 1418 children and young people with JIA by ethnic group and socioeconomic position.

Variable	Ethnic group				IMD group	
	White	Asian	Black	Mixed	Most deprived quintile	All others
N	1272	86	24	36	282	848
**JADAS-71**						
Baseline, mean (95% CI)	13.8 (13.2, 14.4)	13.4 (11.3, 15.6)	17.6 (11.2, 23.9)	12.3 (9.9, 14.7)	12.7 (11.6, 13.7)	13.1 (11.6, 13.7)
6 months, mean (95% CI)	4.9 (4.6, 5.3)	6.6 (4.9, 8.2)	4.8 (2.6, 7.1)	4.4 (2.7, 6.1)	5.1 (4.4, 5.7)	4.7 (4.3, 5.1)
Change, mean (95% CI)	−8.9 (−9.5, -8.3)	−6.8 (−9.0, −4.7)	−12.7 (−19.3, −6.2)	−7.9 (−10.7, −5.2)	−7.7 (−8.7, −6.5)	−8.4 (−9.1, −7.7)
Relative mean change (95% CI)	Reference	2.1 (−0.3, 4.5)	0-3.8 (−8.3, 0.07)	1.0 (−2.7, 4.6)	0.8 (−0.6, 2.2)	Reference
Adjusted relative mean (95% CI)	Reference	1.9 (−0.1, 3.8)	−0.9 (−3.4, 5.2)	−0.5 (−3.2, 2.2)	0.4 (−0.4, 1.3)	Reference
**6-month outcomes**						
MDA, % (95% CI) [*n*]	54 (50, 57) [1150]	43 (31, 56) [77]	51 (25, 77) [19]	61 (42, 81) [30]	52 (45, 59) [254]	55 (51, 60) [738]
ACR-Pedi-30 response	39 (36, 42)	29 (19, 39)	37 (17, 56)	49 (33, 66)	38 (32, 43)	40 (37, 43)
ACR-Pedi-50 response	38 (35, 40)	29 (19, 39)	36 (17, 56)	45 (28, 61)	36 (30, 42)	39 (35, 42)
ACR-Pedi-70 response	34 (31, 37)	21 (12, 30)	33 (14, 52)	37 (20, 53)	31 (26, 37)	36 (33, 39)
ACR-Pedi-90 response	28 (26, 31)	20 (11, 28)	29 (11, 47)	33 (17, 48)	26 (21, 31)	30 (27, 34)

MDA defined as: persistent-oligoarthritis PGA ≤2.5 cm and 0 AJC; Enthesitis-related JIA (ERA) were excluded as MDA is not validated for these patients; all other ILAR subtypes PGA ≤3.4 cm, PGE ≤2 cm and AJC ≤1 [26]. *n* for MDA is shown in square brackets, as it excludes patients with ERA.

IMD: Index of Multiple Deprivation; MDA: minimal disease activity.

The proportion of children and young people who achieved minimal disease activity (MDA) following 6 months of TNFi treatment was similar between ethnic groups and IMD groups (Table 2). The proportions attaining ACR-Pedi-30/50/70/90 responses were also similar across ethnic groups (the latter ranging from 20% to 33% by ethnic groups).

In the multivariable linear regression investigating change in JADAS-71 after 6 months of TNFi treatment (Supplementary Table S7; 1130 patients with complete IMD data available included in the model), neither ethnicity nor IMD were statistically significantly associated with change in JADAS-71 score. The interaction between ethnicity and IMD was not significant.

All ethnic groups and the dichotomized IMD groups showed an improvement in each of AJC, LJC, PGA, PGE, CHAQ, ESR and pain over time (Supplementary Table S8).

### Persistence of TNFi treatment

**Figure 1:**
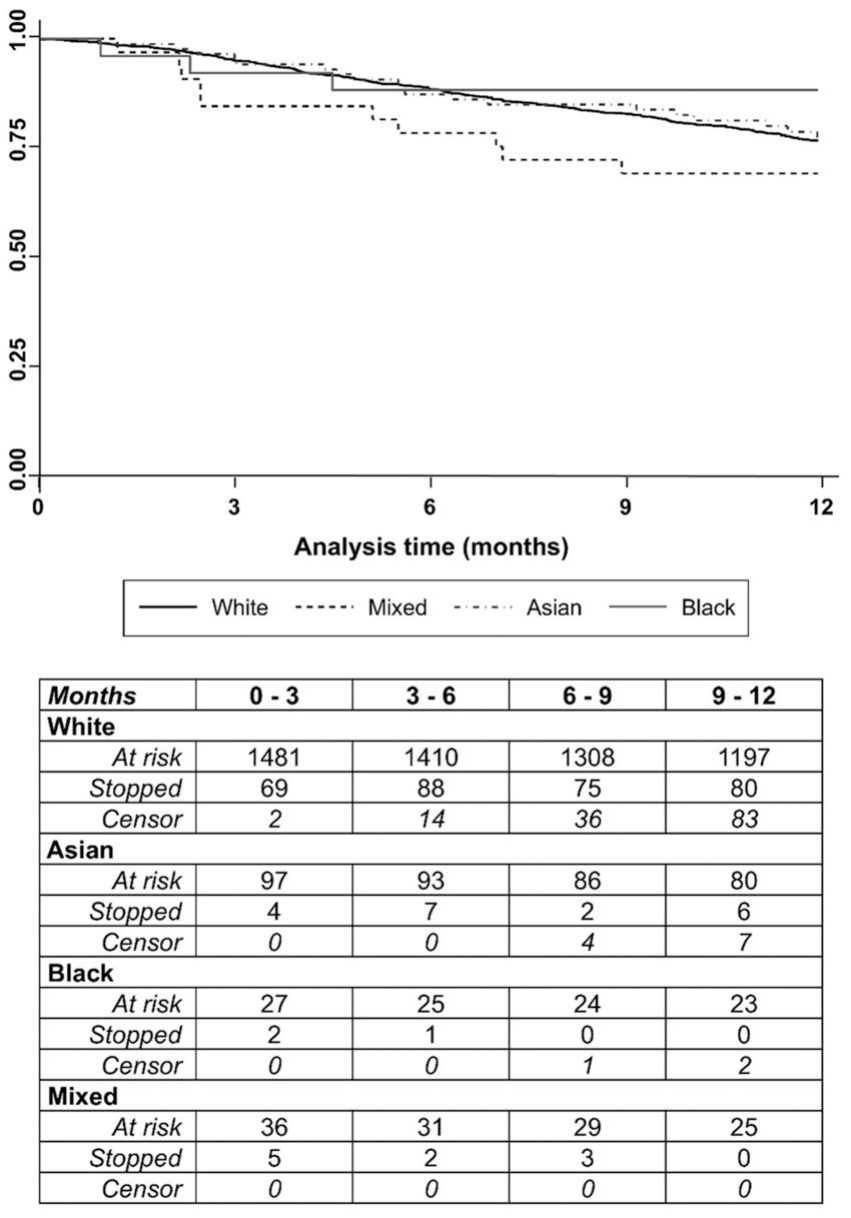
Kaplan–Meier survival curve for first TNFi treatment for 1641 children and young people with JIA by ethnic group: White (*N* = 1481); Asian (*N* = 97); Black (*N* = 27); and Mixed (*N* = 36)

**Figure 2:**
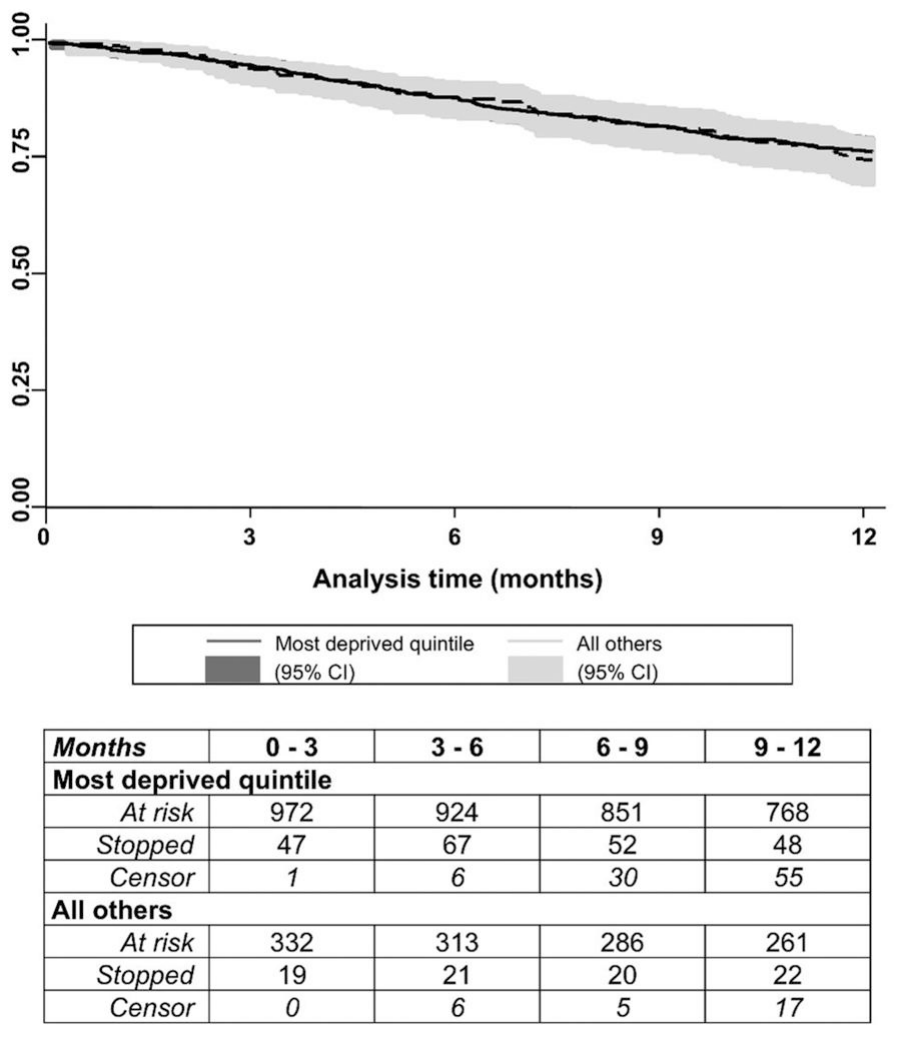
Kaplan–Meier survival curve for first TNFi treatment for 1304 children and young people with JIA by Index of Multiple Deprivation (IMD) group (for whom IMD data were available); those in the most deprived quintile (*N* = 332) compared with all other patients (*N* = 972)

Kaplan–Meier survival curves for TNFi treatment persistence up to 12 months are shown by ethnic group (Fig. 1) and IMD group (Fig. 2). Overall, 67% of patients remained on TNFi at 12 months (95% CI 66%, 68%); this was similar between ethnic groups and IMD groups (Table 3). Median treatment survival was 1.2 years (95% CI 0.8, 2.2 years). The adjusted hazard ratios identified no differences in treatment persistence across ethnic groups, nor between IMD groups. Similar results were seen in an analysis limited to those starting their first TNFi from 2010 onwards (median treatment survival 1.2 years); patients in this sensitivity analysis were similar to the full cohort (Supplementary Table S4).

**Table 3:** Hazard ratio for treatment survival of first TNFi treatment amongst 1641 children and young people with JIA by ethnic group and IMD.

	Ethnic group				IMD group	
	White	Asian	Black	Mixed	Most deprived quintile	All others
**All patients**						
N	1481	97	27	36	332	972
Percentage remaining on TNFi (95% CI)	67 (66, 68)	68 (64, 73)	75 (67, 83)	59 (51, 67)	64 (62, 66)	67 (66, 68)
Unadjusted hazard ratio (95% CI)	Reference	0.96 (0.60, 1.52)	0.50 (0.16, 1.57)	1.46 (0.78, 2.74)	1.07 (0.83, 1.38)	Reference
Adjusted hazard ratio (95% CI)	Reference	0.95 (0.58, 1.56)	0.62 (0.20, 1.93)	1.20 (0.57, 2.55)	1.08 (0.83, 1.37)	Reference
**Sensitivity analysis** ^a^						
N	1013	74	23	31	251	773
Percentage remaining on TNFi (95% CI)	65 (64, 66)	68 (63, 73)	74 (66, 83)	58 (49, 67)	64 (62, 67)	65 (64, 67)
Unadjusted hazard ratio (95% CI)	Reference	0.87 (0.60, 1.26)	0.71 (0.33, 1.49)	1.44 (0.91, 2.28)	1.07 (0.87, 1.31)	Reference
Adjusted hazard ratio (95% CI)	Reference	1.06 (0.71, 1.59)	0.91 (0.41, 2.07)	1.23 (0.72, 2.11)	1.14 (0.92, 1.41)	Reference

Adjusted hazard ratio adjusted for age, gender, grouped ILAR subclass, IMD group and time between diagnosis and commencement of TNFi treatment. ^a^Sensitivity analysis includes only patients starting first TNFi from 2010 onwards.

**Table:** 

Centre	Principal Investigator
(Adult) Broadgreen Hospital	Dr Devesh Mewar
(Adult) Chapel Allerton Hospital	Dr Helen Marzo-Ortego
(Adult) Freeman Hospital	Dr Flora McErlane
(Adult) Manchester Royal Infirmary	Dr Ben Parker
(Adult) Minerva Health Centre (Lancashire)	Dr Elizabeth MacPhie
(Adult) New Cross Hospital	Dr Srinivasan Venkatachalam
(Adult) Pinderfields Hospital	Dr Sharmin Nizam
(Adult) Queens Medical Centre, Nottingham	Dr Ian Gaywood
(Adult) Royal Hallamshire Hospital	Dr Rachel Tattersall
(Adult) Russells Hall Hospital	Dr Niki Erb
(Adult) Stepping Hill Hospital	Dr Abbas Ismail
(Adult) University College London Hospital	Dr Coziana Ciurtin/Dr Debajit Sen
(Adult) University Hospital Aintree	Professor Robert Moots
(Adult) Wishaw General Hospital	Dr Elizabeth Murphy
(Adult) Wrightington Hospital	Dr Easwaradhas Gladston-Chelliah
(Adult) York Hospital	Dr Mark Quinn
Addenbrookes Hospital	Dr Kate Armon
Alder Hey Hospital, Liverpool	Dr Michael Beresford/Dr Liza McCann
Ashford and St Peters Hospital	Dr Gillan Bask/Lorna Walding/Susan Marchant
Basildon Hospital, Essex	Dr Nagui Gendi
Birmingham Children’s Hospital	Dr Eslam Al-Abadi
Bradford Royal Infirmary	Charlene Bass Woodcock
Bristol Royal Hospital for Children	Dr Athimalaipet Ramanan
Cheltenham General Hospital	Dr Louise Walker
Chesterfield Royal Hospital	Dr John Bourne
Crosshouse Hospital, Kilmarnock	Dr Bridget Oates
Derbyshire Children’s Hospital	(site no longer active)
Derriford Hospital, Plymouth	Dr Vicky Ohlsson
Diana Princess of Wales Hospital, Grimsby	Dr David James
Evelina London Children’s Hospital	Dr Nick Wilkinson
George Eliot Hospital	Dr Kathy Bailey
Great North Children’s Hospital Newcastle	Dr Flora McErlane
Great Ormond Street Hospital, London	Dr Ovgu KulCinar
Hinchingbrooke Hospital, Huntingdon	Dr Gill Pountain
King’s Mill Hospital	Dr Mitra
Leeds General Infirmary	Dr Mark Wood
Leicester Royal Infirmary	Dr Arani Sridhar
New Cross Hospital, Wolverhampton	Dr Karen Davies
Norfolk and Norwich University Hospital	Dr Kate Armon
Northampton General Hospital	Dr Rachel Jeffery/Imogen Norton
Nuffield Orthopaedic Centre, Oxford	Dr Kathy Bailey
Peterborough City Hospital	Dr Afraa Al-Sabbagh
Poole Hospital	Dr Fouz Rahmeh
Queen Alexandra Hospital, Portsmouth	Dr Joanne Borbone
Queen Elizabeth Hospital, Woolwich	(site no longer active)
Queen Elizabeth the Queen Mother Hospital, Margate	Dr Alison Leak
Queen’s Medical Centre, Nottingham	Dr Rangaraj Satyapal/Dr Kishore Warrier
Royal Aberdeen Children’s Hospital	Dr Gulshan Malik
Royal Derby Hospital	Dr Richard Bowker
Royal Devon and Exeter Hospital	Dr Nigel Osborne
Royal Hospital for Children & Young People, Edinburgh	Dr Catriona Anderson
Royal Hospital for Children, Glasgow	Dr Jo Walsh
Royal Manchester Children’s Hospital	Dr Alice Chieng
Royal Shrewsbury Hospital	Dr Richard Brough
Royal Stoke University Hospital	Dr Jonathon Packham/Dr Alex Tabor
Sheffield Children’s Hospital	Dr Daniel Hawley
Sheffield Children’s Hospital	Dr Daniel Hawley
Southampton General Hospital	Dr Alice Leahy
Southend University Hospital	Dr Rajesh Rawlani
Staffordshire Rheumatology Centre	Dr Jon Packham
Tayside Children’s Hospital	Dr Jo Walsh
The Great Western Hospital	Dr Rosemary Waller
The Robert Jones & Agnes Hunt Orthopaedic Hospital	Dr Karen Davies
University Hospital Coventry	Dr Willam Coles
University Hospital Wales, Cardiff	Dr Jeremy Camilleri
Worthing Hospital	Dr Lisa Bray

## Discussion

This is the first study specifically investigating changes in disease activity and treatment persistence following first TNFi treatment in children and young people with JIA by ethnic group and socioeconomic position. Overall, following TNFi treatment children and young people with JIA showed improvements in all analysed measures of disease activity. Reassuringly there was no evidence that improvements differed significantly by ethnic group or by socioeconomic position in this analysis. Additionally, duration on first TNFi (used as a surrogate marker of controlled disease) was not associated with ethnic group or socioeconomic position.

While around 61% of children and young people with JIA remain on methotrexate after one year of treatment [21], a significant proportion progress to biologic therapies. TNFi were the first biologic therapies licensed for use in JIA [23], and with their established efficacy and safety profile, remain the most frequently prescribed class of biologic treatments for children and young people with JIA [24]. Whilst previous studies have considered disease outcomes of patients with JIA [12], to date the possible association with social determinants of health have not been considered.

This study has found improvements in disease activity over the first six months, measured by the JADAS-71, for all ethnic groups and IMD groups following TNFi initiation. These improvements took place alongside improvements in individual core outcome variables including active and limited joint counts, physician’s assessment of overall disease activity, patient/parent evaluation of overall wellbeing, functional ability, laboratory inflammatory markers and patient-reported pain. Importantly, these measures at both start of therapy and after 6 months (including change in disease activity), were similar between all ethnic groups and IMD groups. In addition, around half of patients in this study attained minimal disease activity by 6 months after starting treatment with a TNFi, regardless of their ethnic group or IMD group. This is a higher proportion than attained by treatment-naive patients commencing treatment with methotrexate (45%) [21]. The proportion of patients attaining ACR-Pedi was similar between groups; the proportion attaining ACR-Pedi was similar to that following methotrexate [21].

This study is based on data from a national cohort study following patients over time monitoring treatment and disease activity. Whilst it seeks to be broadly representative of the whole UK JIA population, it appears that children and young people of White ethnicity may be over-represented in this analysis. It is not possible to determine whether this is due to selection bias at recruitment, or differences in prescribing patterns and/or treatment uptake. Children from the most deprived groups are slightly over-represented in this analysis (25%, compared with an expected 20% of the population as a whole), and 19% of children and young people with a diagnosis of JIA [25].

Limitations of this study include the relatively small sample size within the Asian, Black and Mixed ethnic groups. These small numbers lead to increased variability and decreased statistical power, limiting our ability to make broader conclusions. Additionally, some outcome measures had a high level of missing data, which may affect the precision of estimates, although the use of multiple imputation sought to minimize the impact of selection bias, and analysis models were adjusted for patient characteristics at the start of TNFi treatment to account for any measured confounding. The analyses did not include an assessment of safety or occurrence of adverse events, which would add to this evidence base. The relatively small number of patients meant that year of starting TNFi had to be grouped into bands, although the sensitivity analysis of drug persistence, restricted to patients starting TNFi after 2010, did not indicate differences compared with whole cohort.

Nonetheless, it appears that in this group of children and young people with JIA, outcomes following TNFi initiation are similar across ethnic and IMD groups. Ethnicity and IMD do not appear to be associated with significant differences in disease activity over time, and there was no evidence that any potential effects of IMD status are moderated significantly by ethnicity or vice versa. Whilst further research to identify potential differences associated with social determinants of health are warranted, the findings of this study—that there is no apparent difference in outcomes following TNFi initiation based on ethnicity and socioeconomic position—should be of reassurance to prescribers and patients starting TNFi.

## Supplementary Material

keag318_Supplementary_Data

## Data Availability

The data underlying this article cannot be shared publicly to maintain the privacy of the individuals who participated in the study. The data that support the findings of this study are available via application to the UK JIA Biologics Register Scientific Steering Committee (via the corresponding author). Restrictions apply to the availability of these data.
