# Supplementary material for: The association between ethnicity, socioeconomic position and outcomes following initiation of TNF inhibitors in juvenile idiopathic arthritis: results from the UK JIA Biologics Register
Source: Rheumatology (Oxford). 2026 Jun 19;65(7):keag318. doi: 10.1093/rheumatology/keag318 (PMC13335643; doi:10.1093/rheumatology/keag318)

**Supplementary tables**

**Supplementary Table S1**: Definition of Minimal Disease Activity (MDA)

| **JIA ILAR subtype** | **Calculation of MDA** |
| --- | --- |
| Persistent-oligoarthritis | PGA ≤ 2.5cm, and AJC=0. |
| Enthesitis-related JIA (ERA) | Excluded – MDA is not validated for ERA. |
| All others | PGA ≤ 3.4cm, and PGE ≤ 2cm, and AJC ≤ 1. |

**Supplementary Table S2**. Modification of ACR-Pedi-30/50/70/90 to take account of low baseline scores.

| **Component** | **Original ACR-Pedi-30** | **Modified ACR-Pedi-30 for analyses** |
| --- | --- | --- |
| PGA | Change in value between baseline and follow-up assessment. | As original, except a score of 0 at both baseline and follow-up was considered to be an improvement of at least 30%; a score of 0 at baseline and ≥0 at follow-up is considered to be a worsening of more than 30%. |
| PGE | Change in value between baseline and follow-up assessment. | As original, except a score of 0 at both baseline and follow-up was considered to be an improvement of at least 30%; a score of 0 at baseline and ≥0 at follow-up is considered to be a worsening of more than 30%. |
| AJC | Change in value between baseline and follow-up assessment. | As original, except a score of 0 at both baseline and follow-up was considered to be an improvement of at least 30%; a score of 0 at baseline and ≥0 at follow-up is considered to be a worsening of more than 30%. |
| LJC | Change in value between baseline and follow-up assessment. | As original, except a score of 0 at both baseline and follow-up was considered to be an improvement of at least 30%; a score of 0 at baseline and ≥0 at follow-up is considered to be a worsening of more than 30%. |
| CHAQ | Change in value between baseline and follow-up assessment. | As original, except a score of 0 at both baseline and follow-up was considered to be an improvement of at least 30%; a score of 0 at baseline and ≥0 at follow-up is considered to be a worsening of more than 30%. |
| ESR | Change in value between baseline and follow-up assessment. | Values <20mm/hr were considered normal. Any change within this normal range, regardless of value, was considered as an improvement.  If follow-up is ≥20mm/hour when baseline had been <20mm, then % worsening is calculated from 20mm/hour. Values ≥20mm at baseline are treated as face value and response/worsening calculated appropriately. |
| ACR Pedi-30 | Proportion of patients improvement between baseline and follow-up assessment in at least three of PGA, PGE, AJC, LJC, CHAQ, ESR; with no more than 1 remaining variable worsening by more than 30%. | Proportion of patients improvement between baseline and follow-up assessment in at least three of PGA, PGE, AJC, LJC, CHAQ, ESR; with no more than 1 remaining variable worsening by more than 30%, using modified variables above. |

PGA - Physician Global Assessment of disease activity. PGE - Patient/parent Global Evaluation of wellbeing. AJC - Active Joint Count. LJC - Limited Joint Count. CHAQ - Childhood Health Assessment Questionnaire (CHAQ). ESR - Erythrocyte Sedimentation Rate. ACR-Pedi-50/70/90 differ from ACR-Pedi-30 in that at least three variables must show improvement of at least 50%, 70% or 90% respectively compared to baseline, with no more than 1 remaining variable worsening by more than 30%.

**Supplementary Table S3**. Proportion, by ethnic group, of children and young with JIA commencing TNFi therapy compared to national population estimates and the estimated proportion of children and young people with JIA.

|  | **White** | **Mixed** | **Asian** | **Black** | **Other** |
| --- | --- | --- | --- | --- | --- |
| England & Wales, under 16s* | 73% | 7% | 12% | 5% | 3% |
| Incident JIA cases** | 83% | 4% | 6% | 3% | 3% |
| This analysis | 90% | 2% | 6% | 2% | 0% |

* England & Wales national population estimates for under 16s from 2011 Census (ONS). ** Proportion of JIA cases by ethnic group(1)

**Supplementary Table S4**. First TNFi for children and young people with JIA by ethnic group and year of starting the drug.

| **Drug** | **Pre 2010** | | | | | **2010-15** | | | | | **2016+** | | | | |
| --- | --- | --- | --- | --- | --- | --- | --- | --- | --- | --- | --- | --- | --- | --- | --- |
|  | **White** | **Asian** | **Black** | **Mixed** | **White** | | **Asian** | **Black** | **Mixed** | **White** | | **Asian** | **Black** | **Mixed** |  |
| **All**, n (%) | 389 (31) | 21 (24) | 4 (17) | 5 (14) | 382 (30) | | 32 (37) | 8 (33) | 20 (56) | 501 (39) | | 33 (38) | 12 (50) | 11 (31) |  |
| **Proportion by drug, %** |  |  |  |  |  | |  |  |  |  | |  |  |  |  |
| Etanercept | 100 | 100 | 100 | 100 | 70 | | 59 | 50 | 70 | 42 | | 45 | 67 | 45 |  |
| Infliximab | 0 | 0 | 0 | 0 | 8 | | 19 | 0 | 20 | 2 | | 0 | 0 | 0 |  |
| Adalimumab | 0 | 0 | 0 | 0 | 22 | | 22 | 50 | 10 | 56 | | 55 | 33 | 55 |  |

**Supplementary Table S5**. Baseline characteristics of the 1,418 children and young people with JIA commencing first TNFi treatment (included in disease activity analysis).

| **Characteristic** | | **Whole cohort** | **Ethnic group** | | | | |  | | **IMD Group** | | |
| --- | --- | --- | --- | --- | --- | --- | --- | --- | --- | --- | --- | --- |
|  |  |  | **White** | **Asian** | **Black** | **Mixed** |  | | **Most deprived quintile** | | **All others** |  |
| n (row %) |  | 1418 | 1272 (90) | 86 (6) | 24 (2) | 36 (3) |  | | 282 (25) | | 848 (75) |  |
| 8 | Male | 474 (33) | 422 (33) | 27 (31) | 11 (46) | 14 (39) |  | | 93 (33) | | 276 (33) |  |
|  | Female | 944 (67) | 850 (67) | 59 (69) | 13 (54) | 22 (61) |  | | 189 (67) | | 572 (67) |  |
| Age at start of first TNFi | Median (IQR) | 11 (7, 14) | 11 (7, 14) | 12 (7, 14) | 12 (8, 14) | 10 (5, 14) |  | | 10 (7, 13) | | 11 (7, 14) |  |
| Disease duration (time between diagnosis and commencement of first TNFi), years | Median (IQR) | 2 (1, 5) | 2 (1, 5) | 2 (1, 4) | 2 (1, 4) | 2 (1, 3) |  | | 2 (1, 4) | | 2 (1, 5) |  |
| TNFi, n (%) | Etanercept | 959 (68) | 865 (68) | 55 (64) | 15 (63) | 24 (67) |  | | 200 (71) | | 517 (61) |  |
|  | Adalimumab | 406 (29) | 364 (29) | 25 (29) | 9 (38) | 8 (22) |  | | 13 (5) | | 35 (4) |  |
|  | Infliximab | 53 (4) | 43 (3) | 6 (7) | 0 (0) | 4 (11) |  | | 69 (24) | | 296 (35) |  |
| Start year of TNFi, n (%) | Pre-2010 | 419 (30) | 389 (31) | 21 (24) | 4 (17) | 5 (14) |  | | 65 (23) | | 171 (20) |  |
|  | 2010-2015 | 442 (31) | 382 (30) | 32 (37) | 8 (33) | 20 (56) |  | | 107 (38) | | 285 (34) |  |
|  | 2016 onwards | 557 (39) | 501 (39) | 33 (38) | 12 (50) | 11 (31) |  | | 110 (39) | | 392 (46) |  |
| Indices of Multiple Deprivation (IMD) quintile, n (%) | 1 – Most deprived | 282 (25) | 233 (23) | 29 (43) | 10 (56) | 10 (34) |  | | .. | | .. |  |
|  | 2 | 188 (17) | 164 (16) | 17 (25) | 6 (33) | <5 |  | | .. | | .. |  |
|  | 3 | 231 (20) | 214 (21) | 10 (15) | <5 | <5 |  | | .. | | .. |  |
|  | 4 | 203 (18) | 188 (19) | <5 | <5 | <5 |  | | .. | | .. |  |
|  | 5 – Least deprived | 226 (20) | 216 (21) | <5 | <5 | 8 (28) |  | | .. | | .. |  |
| ILAR category, n (%) | Persistent oligo | 172 (12) | 148 (12) | 16 (19) | <5 | <5 |  | | 30 (11) | | 122 (14) |  |
|  | Oligo extended | 284 (20) | 269 (21) | <5 | <5 | 6 (17) |  | | 71 (25) | | 157 (19) |  |
|  | Poly RF- | 521 (37) | 469 (37) | 32 (37) | 8 (33) | 12 (33) |  | | 90 (32) | | 321 (38) |  |
|  | Poly RF+ | 136 (10) | 118 (9) | 11 (13) | <5 | <5 |  | | 28 (10) | | 75 (9) |  |
|  | Psoriatic | 89 (6) | 82 (6) | <5 | <5 | <5 |  | | 24 (9) | | 45 (5) |  |
|  | Enthesitis-related | 168 (12) | 148 (12) | 9 (10) | 5 (21) | 6 (17) |  | | 28 (10) | | 110 (13) |  |
|  | Undifferentiated | 48 (3) | 38 (3) | 8 (9) | <5 | <5 |  | | 11 (4) | | 18 (2) |  |
| History of chronic anterior uveitis at start of treatment, n (%) | Yes | 257 (19) | 226 (19) | 18 (22) | 6 (27) | 7 (21) |  | | 43 (16) | | 170 (21) |  |
|  | No | 1074 (81) | 969 (81) | 62 (78) | 16 (73) | 27 (79) |  | | 224 (84) | | 642 (79) |  |

IQR – Interquartile range. Time to registration – time (years) between diagnosis of JIA and registration and commencement of treatment. IMD – Index of Multiple Deprivation.

**Supplementary Table S6**. Proportion of missing data from demographic and core outcome variables of the 1418 children and young people with JIA commencing first TNFi (included in the disease activity analysis).

| **Variable** | **Ethnic Group** | | | |  | **IMD Group** | | |
| --- | --- | --- | --- | --- | --- | --- | --- | --- |
|  | **White** | **Asian** | **Black** | **Mixed** |  | **Most deprived quintile** | **All others** |  |
| Number of patients | 1272 | 86 | 24 | 36 |  | 282 | 848 |  |
| Gender | 0% | 0% | 0% | 0% |  | 0% | 0% |  |
| Age at start of TNFi | 0% | 0% | 0% | 0% |  | 0% | 0% |  |
| Index of Multiple Deprivation (IMD) | 20% | 21% | 25% | 19% |  | 0% | 0% |  |
| ILAR category | 0% | 0% | 0% | 0% |  | 0% | 0% |  |
| History of chronic anterior uveitis at start of treatment | 6% | 7% | 8% | 6% |  | 5% | 4% |  |
| Disease duration | 1% | 5% | 0% | 3% |  | 1% | 2% |  |
|  |  |  |  |  |  |  |  |  |
| **JADAS-71 (calculated)** |  |  |  |  |  |  |  |  |
| Baseline | 51% | 48% | 58% | 36% |  | 54% | 51% |  |
| 6 Months | 60% | 50% | 63% | 44% |  | 60% | 58% |  |
|  |  |  |  |  |  |  |  |  |
| **cJADAS-71 (calculated)** |  |  |  |  |  |  |  |  |
| Baseline | 42% | 29% | 50% | 28% |  | 46% | 42% |  |
| 6 Months | 47% | 36% | 46% | 33% |  | 48% | 45% |  |
|  |  |  |  |  |  |  |  |  |
| **AJC** |  |  |  |  |  |  |  |  |
| Baseline | 4% | 5% | 4% | 3% |  | 3% | 4% |  |
| 6 Months | 18% | 17% | 21% | 14% |  | 13% | 19% |  |
|  |  |  |  |  |  |  |  |  |
| **LJC** |  |  |  |  |  |  |  |  |
| Baseline | 6% | 8% | 4% | 6% |  | 6% | 6% |  |
| 6 Months | 19% | 19% | 21% | 11% |  | 15% | 19% |  |
|  |  |  |  |  |  |  |  |  |
| **PGA** |  |  |  |  |  |  |  |  |
| Baseline | 31% | 21% | 29% | 22% |  | 32% | 32% |  |
| 6 Months | 38% | 26% | 33% | 25% |  | 37% | 36% |  |
|  |  |  |  |  |  |  |  |  |
| **PGE** |  |  |  |  |  |  |  |  |
| Baseline | 29% | 22% | 38% | 17% |  | 32% | 28% |  |
| 6 Months | 37% | 30% | 42% | 25% |  | 38% | 35% |  |
|  |  |  |  |  |  |  |  |  |
| **Pain** |  |  |  |  |  |  |  |  |
| Baseline | 32% | 26% | 38% | 19% |  | 33% | 30% |  |
| 6 Months | 38% | 27% | 46% | 31% |  | 38% | 36% |  |
|  |  |  |  |  |  |  |  |  |
| **CHAQ** |  |  |  |  |  |  |  |  |
| Baseline | 34% | 24% | 33% | 22% |  | 34% | 30% |  |
| 6 Months | 35% | 26% | 33% | 33% |  | 32% | 35% |  |
|  |  |  |  |  |  |  |  |  |
| **ESR** |  |  |  |  |  |  |  |  |
| Baseline | 16% | 20% | 13% | 17% |  | 16% | 16% |  |
| 6 Months | 34% | 34% | 33% | 22% |  | 29% | 35% |  |

cJADAS-71 - clinical Juvenile Arthritis Disease Activity Score (cJADAS-71). AJC - Active Joint Count. LJC - Limited Joint Count. PGA - Physician Global Assessment of disease activity. PGE - Patient/parent Global Evaluation of wellbeing. CHAQ - Childhood Health Assessment Questionnaire (CHAQ). ESR - Erythrocyte Sedimentation Rate. IMD – Index of Multiple Deprivation. MDA – Minimal Disease Activity.

**Supplementary Table S7**. Relative mean change in JADAS-71 between baseline and 6-month follow-up in the 1130 children and young people with JIA treated with first TNFi, by ethnic group and deprivation.

| **Variable** | **Coefficient (95% CI)** |
| --- | --- |
| Ethnic group |  |
| White | Reference |
| Mixed | -0.5 (-3.2, 2.2) |
| Asian | 1.9 (-0.1, 3.8) |
| Black | 0.9 (-3.4, 5.2) |
| IMD |  |
| Most deprived quintile | 0.4 (-0.4, 1.3) |
| All other quintiles | Reference |
| Ethnicity / IMD interaction |  |
| Mixed / Most deprived quintiles | 1.7 (-2.7, 6.1) |
| Asian / Most deprived quintiles | 0.7 (-22, 3.8) |
| Black / Most deprived quintiles | -2.0 (-7.9. 3.9) |
| Age | 0.1 (0.0, 0.2) |
| Female gender | 0.4 (-0.4, 1.3) |
| Disease duration | -0.1 (-0.2, 0.1) |
| Baseline JADAS | -0.9 (-0.9, -0.8) |
| Subgroup of JIA |  |
| Polyarticular | Reference |
| Oligo persistent | -0.3 (-1.4, 0.8) |
| ERA / Psoriatic | 0.1 (-1.0, 1.1) |
| History of uveitis | -0.2 (-1.3, 0.9) |
| Drug |  |
| Etanercept | Reference |
| Infliximab | 0.6 (-1.2, 2.4) |
| Adalimumab | -0.4 (-1.4, 0.5) |
| Year of start of treatment with first TNFi |  |
| <2010 | Reference |
| 2010-15 | 0.2 (-0.9, 1.2) |
| 2016+ | -0.1 (-1.3, 1.0) |
| Use of glucocorticoids at start of TNFi treatment | 0.7 (-0.1, 1.5) |

Number of patients in model: 1130 (for whom both ethnicity and IMD were available). Coefficient is change in JADAS. A negative value indicates a greater improvement in JADAS-71 compared to the reference group. IMD – Index of deprivation, comparing most deprived quintile to all others.

**Supplementary Table S8**. Measures of disease activity at baseline and six months after commencement of first TNFi of the 1,418 children and young people with JIA, by ethnic group and socioeconomic position.

| **Disease activity measure, mean (95% CI)** | **Ethnic Group** | | | | |  | | **IMD Group** | | |
| --- | --- | --- | --- | --- | --- | --- | --- | --- | --- | --- |
|  | **White** | **Asian** | **Black** | **Mixed** |  | | **Most deprived quintile** | | **All others** |  |
| N | 1272 | 86 | 24 | 36 |  | | 282 | | 848 |  |
|  |  |  |  |  |  | |  | |  |  |
| **cJADAS-71** |  |  |  |  |  | |  | |  |  |
| Baseline | 13.1 (12.6, 13.7) | 12.2 (10.2, 14.1) | 16 (10.1, 21.9) | 11.3 (9.1, 13.6) |  | | 11.8 (10.9, 12.8) | | 12.5 (11.8, 13.1) |  |
| 6 Months | 4.8 (4.4, 5.1) | 6.1 (4.5, 7.6) | 4.5 (2.2, 6.7) | 4.1 (2.4, 5.8) |  | | 4.8 (4.2, 5.5) | | 4.5 (4.1, 4.9) |  |
| Change | -8.4 (-9, -7.8) | -6.1 (-8.1, -4.1) | -11.5 (-17.6, -5.5) | -7.2 (-9.8, -4.6) |  | | -7.0 (-8.0, -5.9) | | -7.9 (-8.6, -7.2) |  |
|  |  |  |  |  |  | |  | |  |  |
| **AJC** |  |  |  |  |  | |  | |  |  |
| Baseline | 5.7 (5.3, 6.1) | 5.1 (3.7, 6.5) | 8.2 (3.6, 12.8) | 3.9 (2.5, 5.4) |  | | 4.6 (4.0, 5.2) | | 5.3 (4.8, 5.7) |  |
| 6 Months | 1.2 (1.1, 1.4) | 1.8 (0.9, 2.7) | 0.9 (0.0, 1.9) | 0.7 (0.0, 1.3) |  | | 1.2 (0.9, 1.5) | | 1.1 (0.9, 1.3) |  |
| Change | -4.5 (-4.9, -4.1) | -3.2 (-4.6, -1.9) | -7.3 (-11.8, -2.8) | -3.3 (-4.9, -1.7) |  | | -3.5 (-4.1, -2.8) | | -4.1 (-4.6, -3.7) |  |
|  |  |  |  |  |  | |  | |  |  |
| **LJC** |  |  |  |  |  | |  | |  |  |
| Baseline | 5 (4.6, 5.4) | 5.3 (3.9, 6.8) | 6.1 (2.9, 9.2) | 3.8 (2.1, 5.4) |  | | 4.3 (3.7, 5.0) | | 4.3 (3.9, 4.8) |  |
| 6 Months | 1.8 (1.5, 2.1) | 4 (1.8, 6.1) | 2.9 (0.3, 5.5) | 1.3 (0.0, 2.5) |  | | 1.5 (1.0, 2.0) | | 1.5 (1.2, 1.8) |  |
| Change | -3.2 (-3.6, -2.8) | -1.4 (-3.5, 0.7) | -3.2 (-5.9, -0.5) | -2.5 (-4.2, -0.8) |  | | -2.8 (-3.5, -2.1) | | -2.8 (-3.3, -2.4) |  |
|  |  |  |  |  |  | |  | |  |  |
| **PGA** |  |  |  |  |  | |  | |  |  |
| Baseline | 3.5 (3.3, 3.6) | 3.1 (2.5, 3.6) | 3.6 (2.5, 4.6) | 3.2 (2.5, 4) |  | | 3.2 (2.9, 3.5) | | 3.4 (3.2, 3.6) |  |
| 6 Months | 1.2 (1.1, 1.3) | 1.4 (0.9, 1.8) | 1.0 (0.3, 1.8) | 1.0 (0.5, 1.5) |  | | 1.1 (0.9, 1.3) | | 1.1 (0.9, 1.2) |  |
| Change | -2.3 (-2.5, -2.1) | -1.7 (-2.3, -1.1) | -2.5 (-3.8, -1.2) | -2.2 (-3, -1.4) |  | | -2.1 (-2.4, -1.7) | | -2.3 (-2.5, -2.1) |  |
|  |  |  |  |  |  | |  | |  |  |
| **PGE** |  |  |  |  |  | |  | |  |  |
| Baseline | 3.9 (3.7, 4.1) | 4.0 (3.4, 4.7) | 4.2 (2.7, 5.7) | 4.1 (3.2, 5.1) |  | | 4.0 (3.6, 4.4) | | 3.8 (3.6, 4.0) |  |
| 6 Months | 2.3 (2.2, 2.5) | 2.9 (2.2, 3.5) | 2.5 (1.2, 3.8) | 2.4 (1.4, 3.4) |  | | 2.6 (2.2, 2.9) | | 2.3 (2.1, 2.5) |  |
| Change | -1.6 (-1.8, -1.3) | -1.2 (-1.9, -0.4) | -1.7 (-3.4, 0.0) | -1.7 (-2.9, -0.5) |  | | -1.4 (-1.9, -1.0) | | -1.4 (-1.7, -1.2) |  |
|  |  |  |  |  |  | |  | |  |  |
| **Pain** |  |  |  |  |  | |  | |  |  |
| Baseline | 4.1 (4.0, 4.3) | 3.9 (3.2, 4.5) | 4.0 (2.7, 5.4) | 4.0 (3.0, 5.0) |  | | 4.2 (3.8, 4.6) | | 4.0 (3.8, 4.2) |  |
| 6 Months | 2.5 (2.3, 2.6) | 2.7 (2.1, 3.3) | 1.9 (0.7, 3.0) | 2.3 (1.4, 3.3) |  | | 2.6 (2.2, 2.9) | | 2.4 (2.2, 2.6) |  |
| Change | -1.7 (-1.9, -1.5) | -1.1 (-1.9, -0.4) | -2.2 (-3.7, -0.6) | -1.7 (-3, -0.4) |  | | -1.6 (-2.1, -1.2) | | -1.6 (-1.8, -1.3) |  |
|  |  |  |  |  |  | |  | |  |  |
| **CHAQ** |  |  |  |  |  | |  | |  |  |
| Baseline | 0.9 (0.9, 1.0) | 1.0 (0.8, 1.2) | 1.0 (0.6, 1.4) | 1.0 (0.7, 1.2) |  | | 1.0 (0.9, 1.1) | | 0.9 (0.8, 0.9) |  |
| 6 Months | 0.6 (0.6, 0.7) | 0.8 (0.6, 1.0) | 0.7 (0.3, 1.0) | 0.6 (0.4, 0.9) |  | | 0.7 (0.6, 0.8) | | 0.6 (0.5, 0.6) |  |
| Change | -0.3 (-0.3, -0.3) | -0.2 (-0.4, 0.0) | -0.3 (-0.7, 0.0) | -0.3 (-0.6, -0.1) |  | | -0.3 (-0.4, -0.2) | | -0.3 (-0.3, -0.2) |  |
|  |  |  |  |  |  | |  | |  |  |
| **ESR** |  |  |  |  |  | |  | |  |  |
| Baseline | 18.4 (17.1, 19.7) | 26.4 (20.2, 32.7) | 28.2 (14.1, 42.3) | 22.4 (13.8, 31) |  | | 20.5 (17.3, 23.8) | | 17.3 (15.8, 18.8) |  |
| 6 Months | 9.7 (8.9, 10.4) | 16.1 (12.0, 20.3) | 13.8 (7.2, 20.5) | 11.9 (7.3, 16.4) |  | | 10.6 (8.8, 12.3) | | 9.4 (8.5, 10.2) |  |
| Change | -8.7 (-10, -7.5) | -10.3 (-16.1, -4.5) | -14.4 (-28.6, -0.2) | -10.5 (-18.4, -2.6) |  | | -10.0 (-13.2, -6.8) | | -7.9 (-9.4, -6.4) |  |

cJADAS-71 - clinical Juvenile Arthritis Disease Activity Score (cJADAS-71). AJC - Active Joint Count. LJC - Limited Joint Count. PGA - Physician Global Assessment of disease activity. PGE - Patient/parent Global Evaluation of wellbeing. CHAQ - Childhood Health Assessment Questionnaire (CHAQ). ESR - Erythrocyte Sedimentation Rate. IMD – Index of Multiple Deprivation.


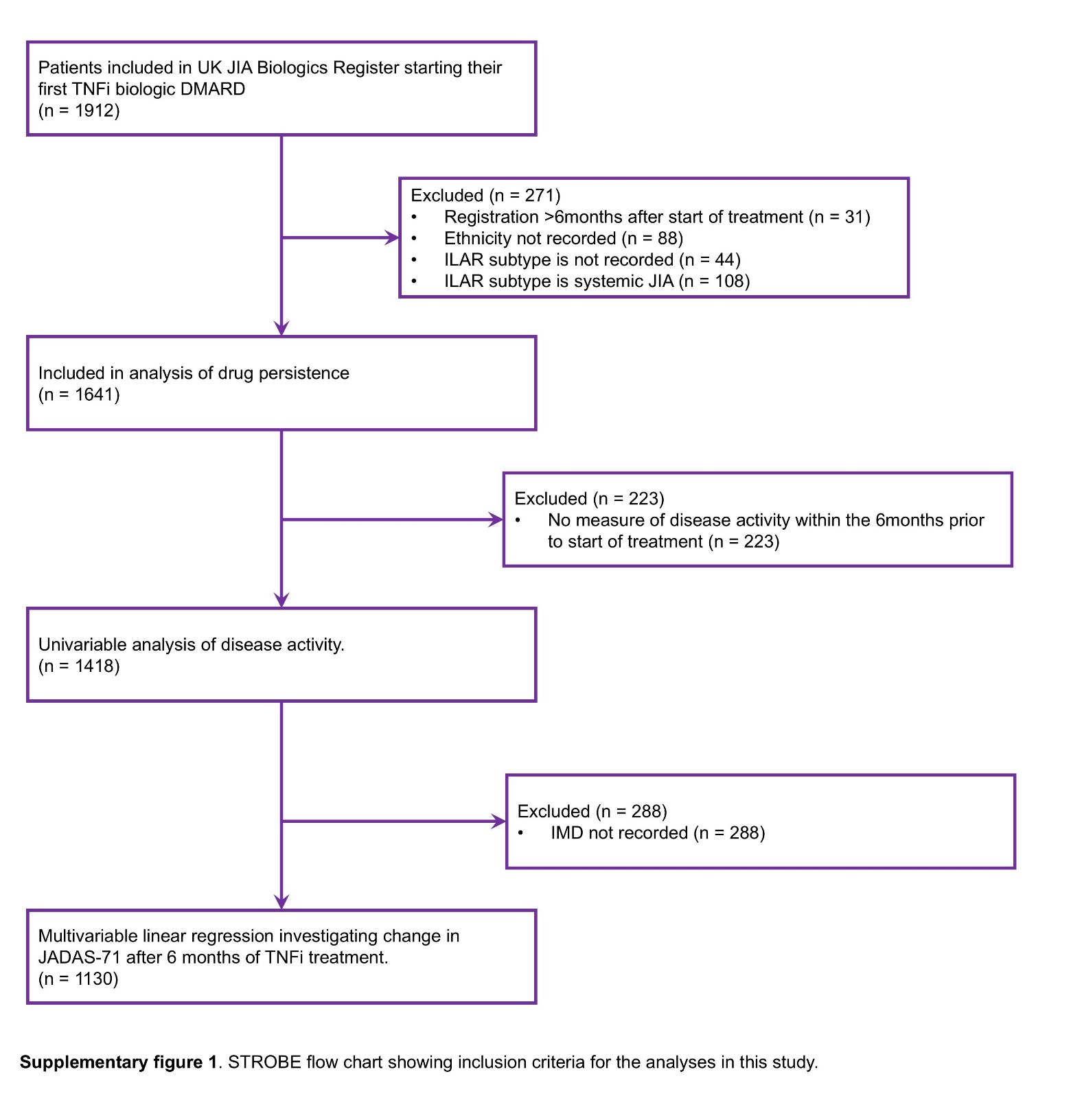

Supplement: keag318_Supplementary_Data [file keag318_supplementary_data.docx]
